# Macrophage expression of E3 ubiquitin ligase Grail protects mice from lipopolysaccharide-induced hyperinflammation and organ injury

**DOI:** 10.1371/journal.pone.0208279

**Published:** 2018-12-20

**Authors:** Chih-Chin Shih, Pei-Yao Liu, Jye-Hann Chen, Mei-Hui Liao, Chih-Ming Hsieh, Shuk-Man Ka, Chin-Chen Wu, Hui-Tsu Lin, Ti-Hui Wu, Ying-Chuan Chen

**Affiliations:** 1 Department of Pharmacology, National Defense Medical Center, Taipei, R.O.C., Taiwan; 2 Department of Physiology & Biophysics, National Defense Medical Center, Taipei, R.O.C., Taiwan; 3 Division of Thoracic Surgery, Department of Surgery, Taichung Armed Force General Hospital, Taichung, R.O.C., Taiwan; 4 Graduate Institute of Aerospace and Undersea Medicine, National Defense Medical Center, Taipei, R.O.C., Taiwan; 5 Institute of Preventive Medicine, National Defense Medical Center, New Taipei City, R.O.C., Taiwan; 6 Division of Thoracic Surgery, Department of Surgery, Tri-Service General Hospital, National Defense Medical Center, Taipei, R.O.C., Taiwan; Max Delbruck Centrum fur Molekulare Medizin Berlin Buch, GERMANY

## Abstract

Multiple organ dysfunction caused by hyperinflammation remains the major cause of mortality during sepsis. Excessive M1-macrophage activation leads to systemic inflammatory responses. Gene related to anergy in lymphocytes (Grail) is regarded as an important regulator of T cells that functions by diminishing cytokine production. However, its role in regulating macrophage activation and organ injury during sepsis remains unclear. Our aim was to examine the effects of Grail on macrophage reactivity and organ injury in endotoxemic animals. Wild-type and Grail knockout mice were injected with vehicle or *Escherichia coli* lipopolysaccharide and observed for 24 h. Changes in blood pressure, heart rate, blood glucose, and biochemical variables were then examined. Moreover, levels of neutrophil infiltration, MMP-9, and caspase 3 were analyzed in the lungs of animals. The expression of pro-inflammatory cytokines in J774A, RAW264.7, and primary peritoneal macrophages stimulated with LPS were also assessed in the presence or absence of Grail. Results indicated that loss of Grail expression enhances the induction of pro-inflammatory cytokines in J774A, RAW264.7, and primary peritoneal macrophages treated with LPS. Furthermore, LPS-induced macrophage hyperactivation was alleviated by ectopic Grail overexpression. *In vivo* studies showed that Grail deficiency exacerbates organ damage in endotoxemic animals. Levels of neutrophil infiltration, MMP-9, and caspase 3 were significantly increased in the lungs of Grail-deficient endotoxemic mice. Thus, these results suggest that Grail contributes to the attenuation of hyperinflammation caused by activated macrophages and prevents organ damage in endotoxemic mice. We suggest that Grail signaling could be a therapeutic target for endotoxemia.

## Introduction

Sepsis and septic shock are still major challenges in intensive care units worldwide. Dysregulated immune responses during sepsis result in the excessive release of pro-inflammatory cytokines and mediators [1, 2]. Systemic inflammation leads to multiple organ dysfunction and a high incidence of mortality [3]. Thus, the identification of factors that can be targeted to mitigate inflammation during sepsis is needed.

The overproduction of inflammatory mediators leads to subsequent tissue damage and multiple organ dysfunction. Macrophages play an important role in innate immunity and inflammatory diseases. Activation of Toll-like receptors (TLRs) on macrophages by inflammatory factors (e.g. lipopolysaccharide, LPS) increases the secretion of various inflammatory cytokines to eradicate the invading organisms [4, 5]. Further, severe activation of M1-macrophages often results in the development systemic inflammatory responses, multiple organ failure, and shock [6, 7]. During the early phase of sepsis, activated M1-macrophages cause hyper-inflammation through the production of pro-inflammatory cytokines and antigen-presenting functions [8]. This altered signaling in macrophages is involved in organ injury during sepsis.

Gene related to anergy in lymphocytes (Grail) has been identified as an important regulator of T cell unresponsiveness, and functions by abrogating the expression of cytokines; deletion of Grail in mice leads to the loss of the anergic phenotype [9, 10]. In response to viral infection, Grail deficiency in mice leads to severe mortality and morbidity by via the control of TBK1 activation [11]. In addition, Grail has been reported to mediate p53-dependent cell cycle arrest and apoptosis by targeting this protein for degradation [12]. Our previous study also demonstrated that Grail interacts with PPAR-γ, thereby regulating adipogenesis and diet-induced obesity [13]. This evidence reveals that Grail has multiple physiological functions in addition to T cell anergy.

A recent study demonstrated the correlation between Grail expression and CD4 T cell unresponsiveness in septic mice [14]. However, the role of this protein in the regulation of LPS-induced macrophage activation and organ injury has not been fully studied *in vivo*. Therefore, we compared macrophage reactivity and organ injury between wild-type and Grail knockout endotoxemic mice to characterize the functional effect of Grail in sepsis.

## Material and methods

### Cell lines

J774A and RAW264.7 cells were cultured in DMEM (Invitrogen) supplemented with 10% FBS (Invitrogen). To isolate peritoneal macrophages, WT and Grail KO mice were injected with 1 ml of 3% Brewer thioglycollate medium into the peritoneal cavity for 4 days. Then, 10 ml of PBS was administrated to the peritoneal cavity of the mouse, and peritoneal fluid was collected into a 50-ml tube and centrifuged to obtain the peritoneal exudate cells. Finally, the supernatant was removed, and cell pellet was resuspended in DMEM.

### Viral constructs and infections

For Grail knockdown experiments, Grail shRNA oligonucleotides were cloned into the pSIREN-Retro-Q empty plasmid (Clontech; Grail shRNA target sequence: 5′-gaggcatccaagtcacaatgg-3′). For Grail overexpression experiments, *Grail* cDNA was cloned into the pQCXIP plasmid (Clontech). For retrovirus production, these plasmids were transfected into GP2-293 cells using TransIT-LT1 according to the protocol published on the Clontech website. The cells were infected with the viruses and then treated with 2 μg/ml puromycin to select and establish stable cell lines. The AAV Helper Free shRNA Expression System was also used according to manufacturer’s instructions (Cell Biolabs). Grail shRNA oligonucleotides were cloned into the pAAV-U6-GFP plasmid (Grail shRNA target sequence: 5′-gaggcatccaagtcacaat-3′). The pAAV-U6-GFP empty vector was used as a control. AAV production and infection were generated according to standard protocols (Cell Biolabs). Cells were stimulated with LPS (100 ng/ml) for 3 h in the presence or absence of Grail.

### Ethics statement

This study was carried out in strict accordance with the recommendations in the Guide for the Care and Use of Laboratory Animals of the National Institutes of Health. The protocol was approved by the Institutional Animal Care and Use Committee of National Defense Medical Center (Taipei, R.O.C., Taiwan) (Permit Number: IACUC-17-020). All surgery was performed under sodium pentobarbital anesthesia, and all efforts were made to minimize suffering.

### Animal ethics and experimental protocols

The animal studies were permitted by the Institutional Animal Care and Use Committee of National Defense Medical Center (Taipei, R.O.C., Taiwan) (Permit Number: IACUC-17-020). We followed the guidelines of humane endpoints and animals were euthanized by overdosed pentobarbital at the end of the experiments. The signs of dyspnea, cyanosis, weight loss, severe diarrhea, seizures, extremity paralysis, dehydration, abnormal posture, and hypothermia were used to determine when the animals should be euthanized. We evaluated animal health every 2 h, and no unexpected deaths were observed in this study. The anesthetic drug was used to decrease the distress and suffering of animals before any stressful process. Male WT and Grail^−/−^ mice (10–20 weeks of age) were generated with a C57BL/6 background as described previously [13].

The mice were randomly assigned into four groups as follows: (i) WT mice receiving saline at time 0 (WT), (ii) WT mice receiving *Escherichia coli* LPS (LPS, 10 mg/kg) at time 0 (WT+LPS), (iii) Grail^−/−^ mice receiving saline at time 0 (Grail^−/−^), and (iv) Grail^−/−^ mice receiving LPS (10 mg/kg) at time 0 (Grail^−/−^+LPS). The *in vivo* examinations were performed for 24 h. The body weight, systolic blood pressure (SBP), and heart rate (HR) of mice were recorded at the beginning and the end of the experiments. In addition, mice were sacrificed to collect blood and lung samples 24 h after saline or LPS administration. Blood glucose and plasma lactate dehydrogenase (LDH) and creatinine (CRE) levels were also examined. Moreover, histopathology and the expression of MMP-9 and caspase-3 in the lungs of animals were analyzed.

### Hemodynamics detection

We used an MK-2000A blood pressure monitor (Muromachi Kikai, Tokyo, Japan) to detect baseline SBP and HR and those 24 h after saline or LPS administration. In addition, 10 μl of whole blood was taken to evaluate the glucose levels using a One Touch II blood glucose monitoring system (Lifescan, Milpitas, CA, USA).

### Organ injury assessment

Blood samples obtained from cardiac puncture were centrifuged at 16,000 × *g* for 2 min to analyze biochemical variables 24 h after saline or LPS administration. All biochemical variables were measured using a Fuji DRI-CHEM 3030 (Fuji Photo Film, Tokyo, Japan). The degree of organ injury was assessed based on increases in plasma levels of LDH and CRE.

### Histopathologic analysis

Lung specimens were harvested and fixed in buffered formaldehyde 24 h after saline or LPS administration. The fixed lungs were then dehydrated, embedded, and stained with hematoxylin and eosin. Histopathologic changes in the lung were evaluated according to the levels of polymorphonuclear neutrophil (PMN) infiltration. Each lung section was assessed by a pathologist, and the infiltration index was given a score from 0 (minimal) to 5 (maximal).

### Real-time PCR

RNA was isolated using TRIzol reagent (Sigma-Aldrich, St. Louis, MO, USA). cDNA was synthesized from 2 μg of total RNA using Epicentre MMLV. Real-time PCR was conducted using an Applied Biosystems 7500 Real-Time PCR system and the IQ2 FAST Q-PCR kit. Primer sequences are shown in S1 Table.

### Enzyme linked immunosorbent assay

The IL-1β and inducible nitric oxide synthase (iNOS) levels in cell lysates were measured with the enzyme linked immunosorbent assay kit (USCN, Hubei Province, China) according to the manufacturer’s instructions.

### Immunoblotting

Macrophages and lung tissues were lysed in RIPA buffer with protease inhibitors. Twenty micrograms of protein was mixed with SDS sample buffer, separated using an SDS-gel, and transferred onto a nitrocellulose membrane. Then, the membranes were probed with anti-iNOS IgG (13120, Cell Signaling, USA), anti-cleaved caspase-3 IgG (9661, Cell Signaling, USA) or anti-actin IgG (MAb1501, Chemicon, USA). Protein levels were quantified using ImageJ software (National Institutes of Health, Bethesda, MD, USA).

### Statistical analysis

Graphing and statistical analyses of data were performed using GraphPad Prism 7 (GraphPad Software). All data are expressed as the mean ± standard error. To compare multiple data sets, one-way or two-way analysis of variance (ANOVA) with multiple comparative analysis was used. To analyze two data sets, an unpaired two-tailed Student's t test was used. *P* values ≤ 0.05 were considered statistically significant.

## Results

### Loss of Grail potentiates the expression of pro-inflammatory cytokines in LPS-stimulated J774A cells

**Fig 1:**
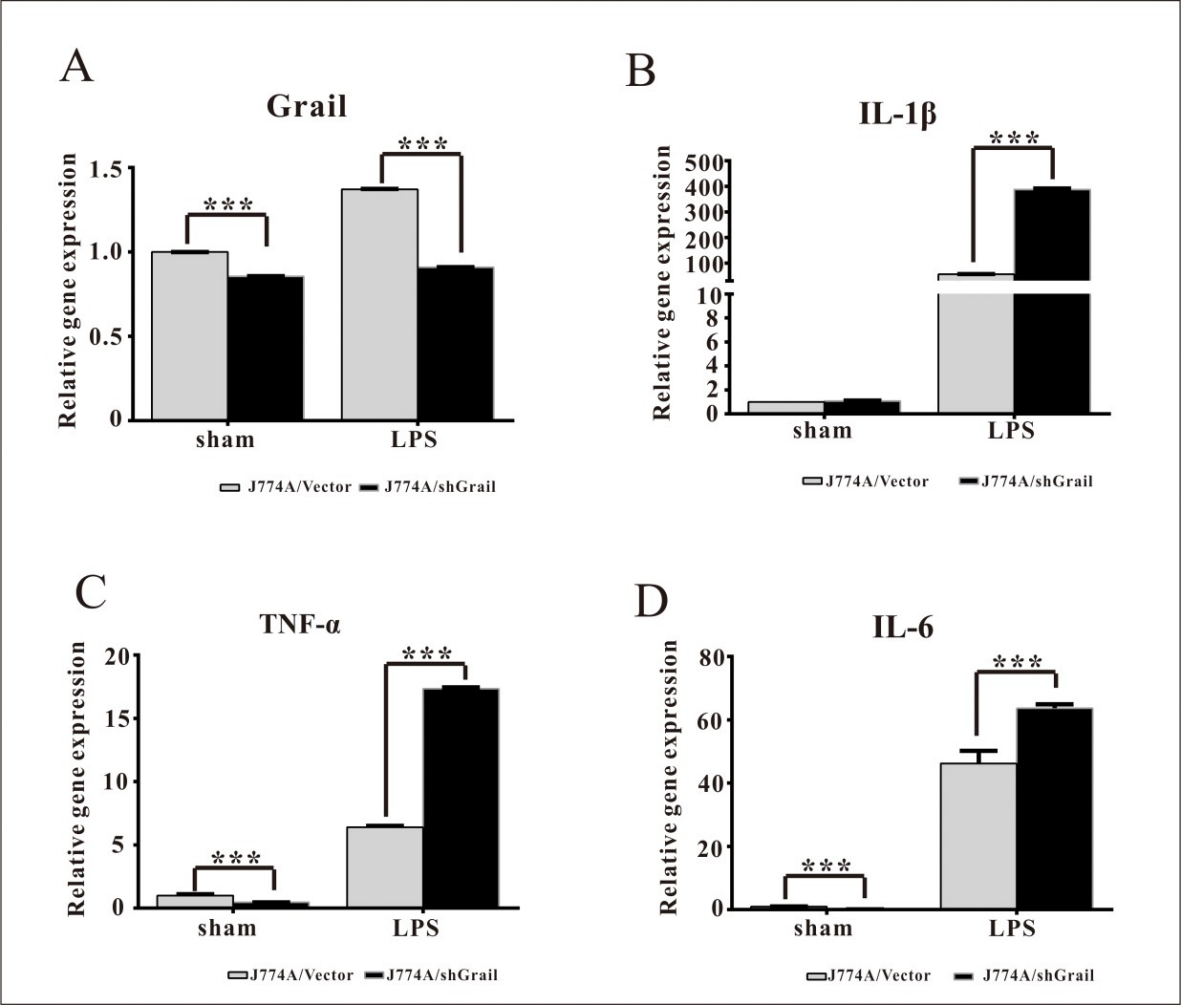
Grail suppresses the expression of pro-inflammatory cytokines in LPS-stimulated J774A cells. The mRNA expression of (A) *Grail*, (B) *IL-1β*, (C) *TNF-α*, and (D) *IL-6* was assessed in J774A/Vector and J774A/shGrail cells after LPS stimulation (3 h; 100 ng/ml). These data are presented as mean ± SEM. A student’s *t-*test was used to assess statistical significance. ***P < 0.001.

To investigate the effects of Grail on the induction of cytokines from macrophage cells, we used shGrail to silence its expression in J774A cells (Fig 1A). LPS treatment resulted in increases in the expressions of *IL-1β*, *TNF-α*, and *IL-6* mRNA in J774A cells (Fig 1B–1D). *IL-1β*, *TNF-α*, and *IL-6* expression levels in J774A/shGrail cells were found to be higher than those in the J774A/Vector cells in the presence of LPS (Fig 1B–1D). This suggests that knockdown of Grail enhances IL-1β, TNF-α, and IL-6 expression in LPS-stimulated J774A cells.

### Grail overexpression attenuates the expression of pro-inflammatory cytokines in LPS-stimulated RAW264.7 cells

**Fig 2:**
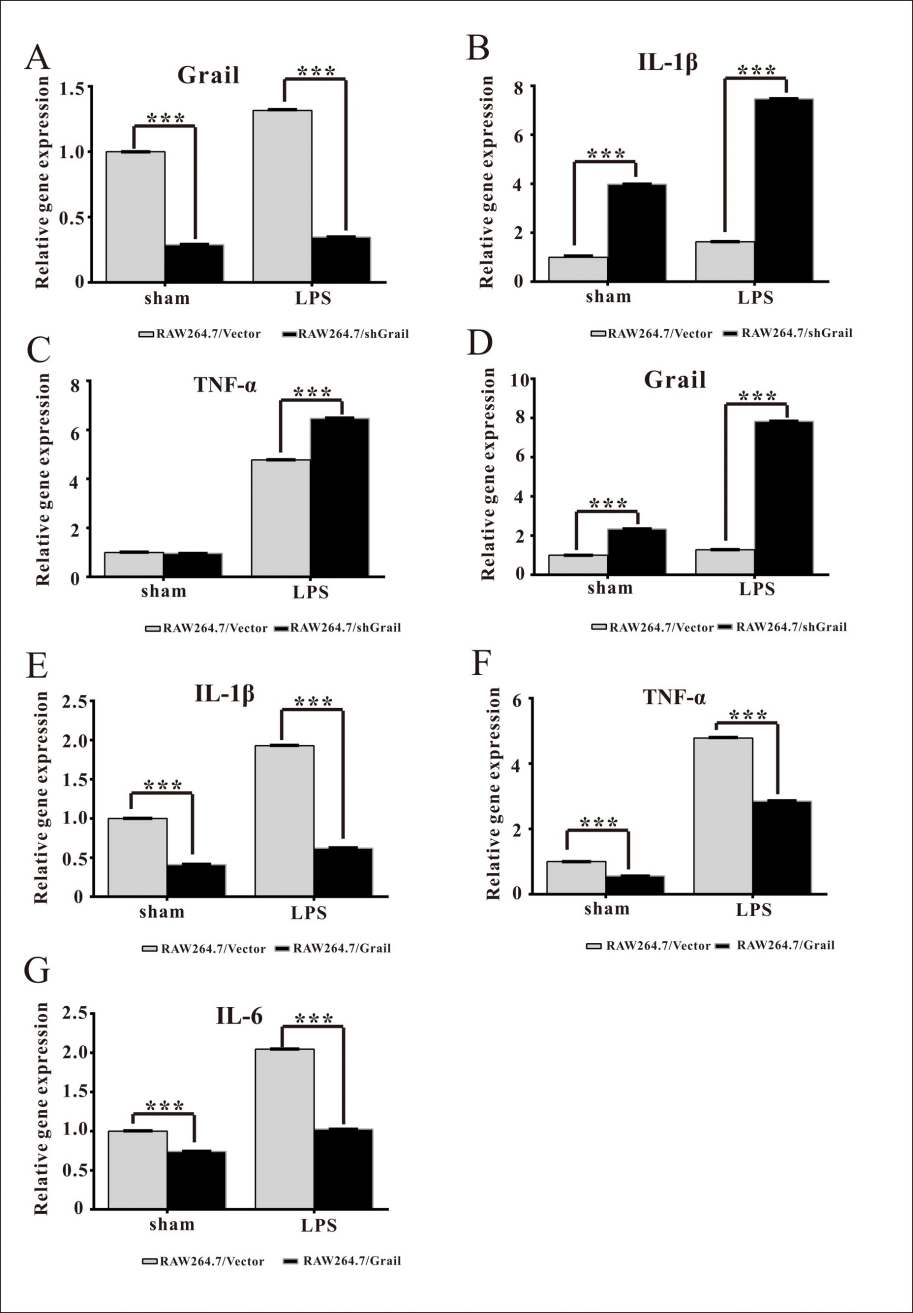
Grail suppresses the expression of pro-inflammatory cytokines in LPS-stimulated RAW264.7 cells. The mRNA expression of (A) *Grail*, (B) *IL-1β*, and (C) *TNF-α* was evaluated in RAW264.7/Vector and RAW264.7/shGrail cells after LPS stimulation (3 h; 100 ng/ml). In contrast, the effects of Grail overexpression on the mRNA expression of (D) *Grail*, (E) *IL-1β*, (F) *TNF-α*, and (G) *IL-6* were examined in RAW264.7 cells after LPS stimulation (3 h; 100 ng/ml). These data are presented as the mean ± SEM. A student’s *t-*test was used to assess statistical significance. ***P < 0.001.

To further investigate the effects of Grail on the LPS-induced activation of macrophages, we silenced and overexpressed Grail in RAW264.7 cells (Fig 2A and 2D). LPS increased IL-1β and TNF-α expression levels in RAW264.7 cells (Fig 2B and 2C). Further, these increases were significantly enhanced by Grail knockdown in RAW264.7 cells (Fig 2B and 2C). In addition, we established Grail-overexpressing stable RAW264.7 cell lines using the retroviral-mediated expression system (Fig 2D). Overexpression of Grail obviously ameliorated *IL-1β*, *TNF-α*, and *IL-6* expression levels in the RAW264.7 cells treated with LPS (Fig 2E–2G). These results indicate that Grail overexpression could attenuate the induction of pro-inflammatory cytokines in LPS-stimulated macrophages.

### Deletion of Grail augments the production of pro-inflammatory cytokines in primary peritoneal macrophages treated with LPS

**Fig 3:**
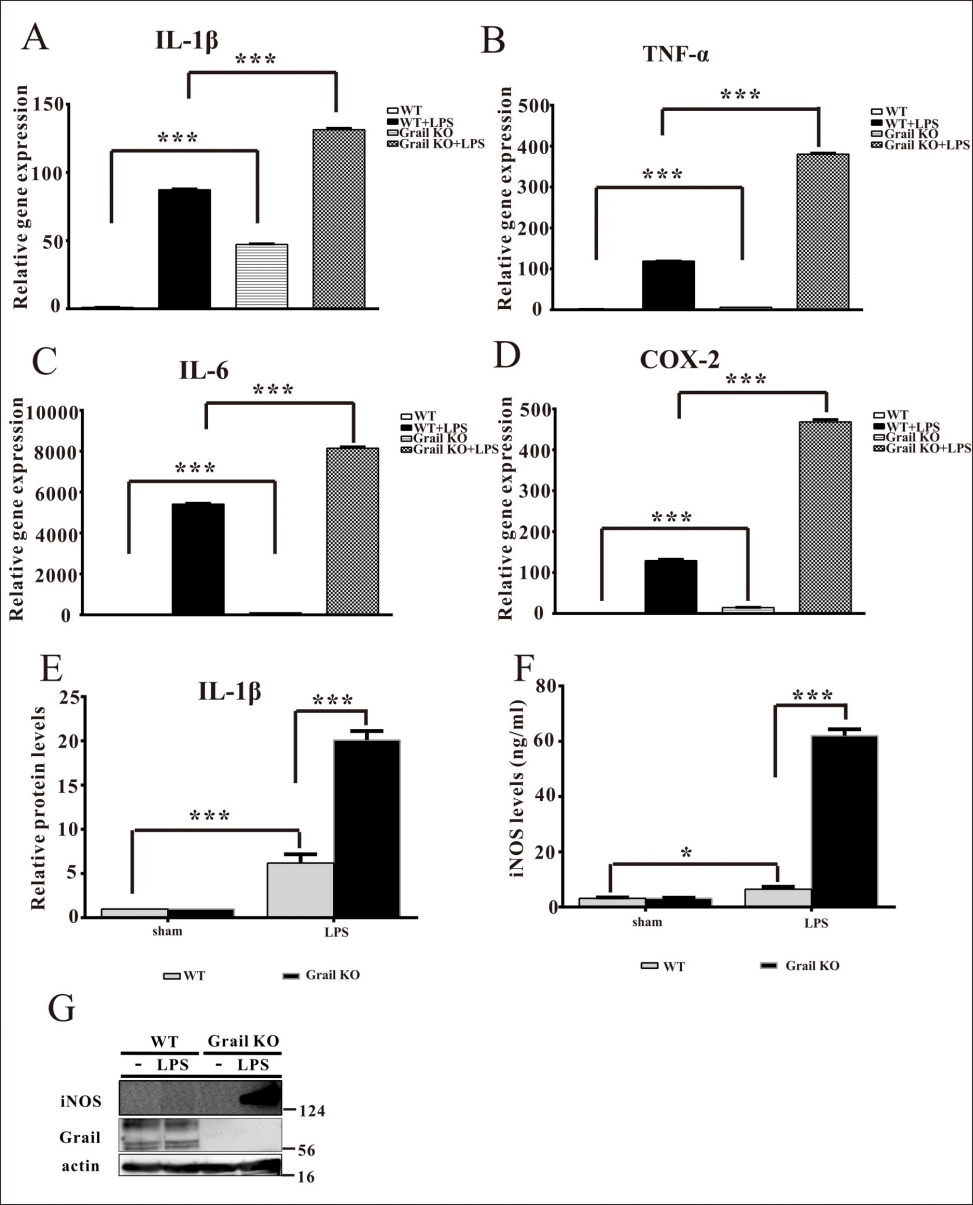
Grail diminishes the expression of pro-inflammatory cytokines in primary peritoneal macrophages treated with LPS. The mRNA expression of (A) *IL-1β*, (B) *TNF-α*, (C) *IL-6*, and (D) *COX-2* was measured in primary peritoneal macrophages isolated from WT and Grail KO mice after LPS stimulation (3 h; 100 ng/ml). The protein expression of (E) IL-1β, (F and G) iNOS was determined in primary peritoneal macrophages isolated from WT and Grail KO mice after LPS stimulation (18 h; 100 ng/ml). These data are presented as the mean ± SEM. A student’s *t-*test was used to assess statistical significance. ***P < 0.001.

To confirm the function of Grail with respect to the activation of macrophages, we isolated primary peritoneal macrophages from WT and Grail KO mice. The expression levels of *IL-1β*, *TNF-α*, *IL-6*, and *COX-2* in primary peritoneal macrophages of Grail KO mice were higher than those in primary peritoneal macrophages of WT mice (Fig 3A–3E). Furthermore, LPS administration resulted in increases in the expression of *IL-1β*, *TNF-α*, *IL-6*, and *COX-2* in primary peritoneal macrophages (Fig 3A–3E). Further, *IL-1β*, *TNF-α*, *IL-6*, and *COX-2* expression levels in the primary peritoneal macrophages of Grail KO mice were higher than those in the primary peritoneal macrophages of WT mice in the presence of LPS (Fig 3A–3E). In addition, the protein levels of M1 macrophage marker iNOS in primary peritoneal macrophages of Grail KO mice were higher than those in primary peritoneal macrophages of WT mice in the presence of LPS (Fig 3F and 3G). These data reveal that Grail plays an important role in inhibiting the production of pro-inflammatory cytokines from activated macrophages.

### Effects of Grail deletion on organ injury in endotoxemic mice

**Fig 4:**
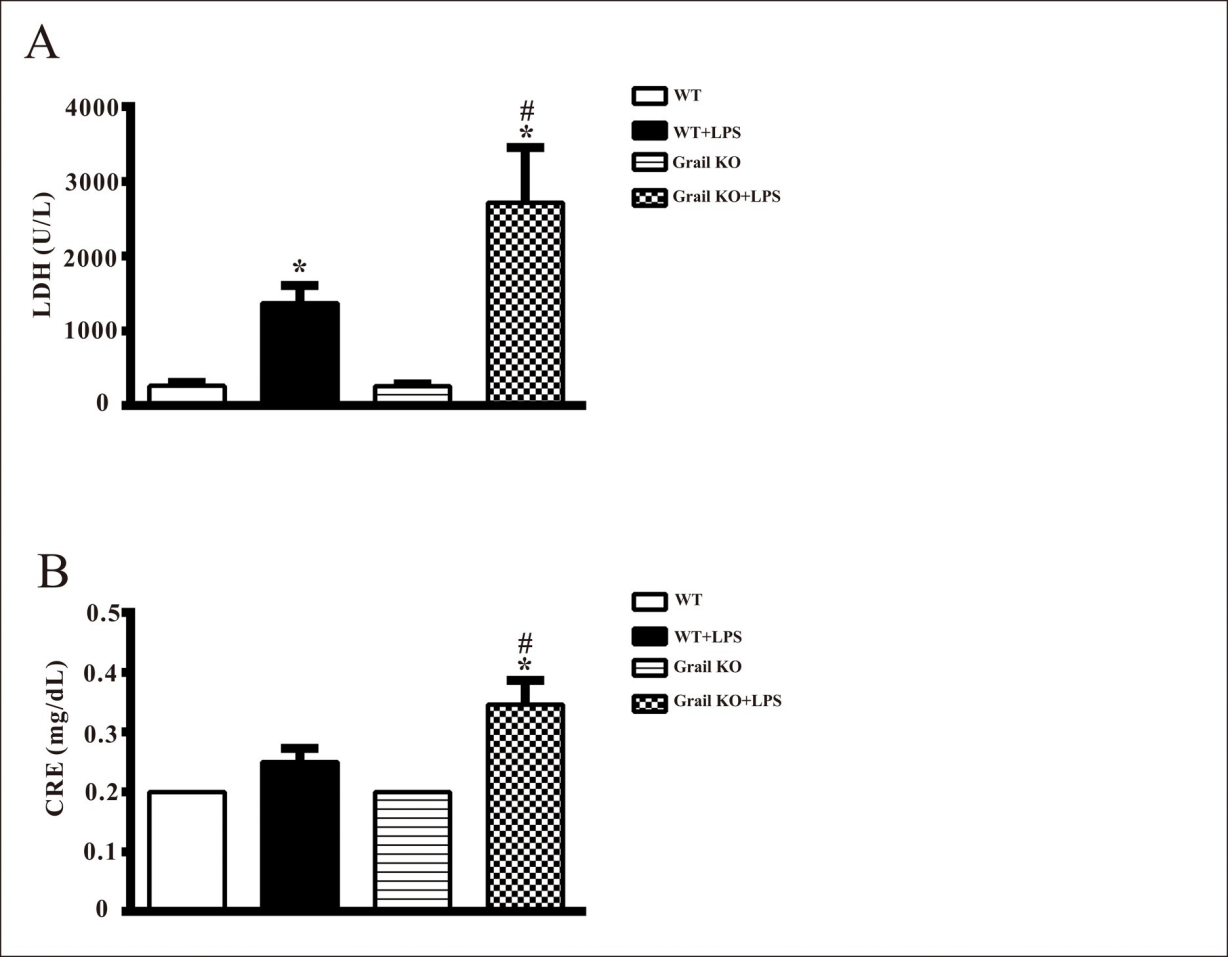
**Effect of Grail deletion on plasma levels of (A) lactate dehydrogenase (LDH) and (B) creatinine (CRE) in mice treated with LPS.** Data are shown for WT mice administered saline at time 0 (WT, n = 8), WT mice administered LPS at time 0 (WT+LPS, n = 10), Grail KO mice administered saline at time 0 (Grail KO, n = 8), and Grail KO mice administered LPS at time 0 (Grail KO+LPS, n = 11). The data are presented as mean ± SEM. A one-way ANOVA with a Newman-Keuls post hoc test was used to evaluate statistical significance. *P < 0.05, LPS versus without LPS; ^#^*P* < 0.05, Grail KO+LPS versus WT+LPS.

There were no significant differences in plasma LDH and CRE levels between WT and Grail^−/−^ groups (Fig 4). The injection of LPS elicited significant increases in plasma levels of LDH and CRE at 24 h in WT and Grail^−/−^ mice (Fig 4). However, increases in LDH and CRE triggered by LPS at 24 h were significantly augmented by Grail deletion (Fig 4).

### Effects of Grail deletion on neutrophil infiltration in the lungs of endotoxemic mice

**Fig 5:**
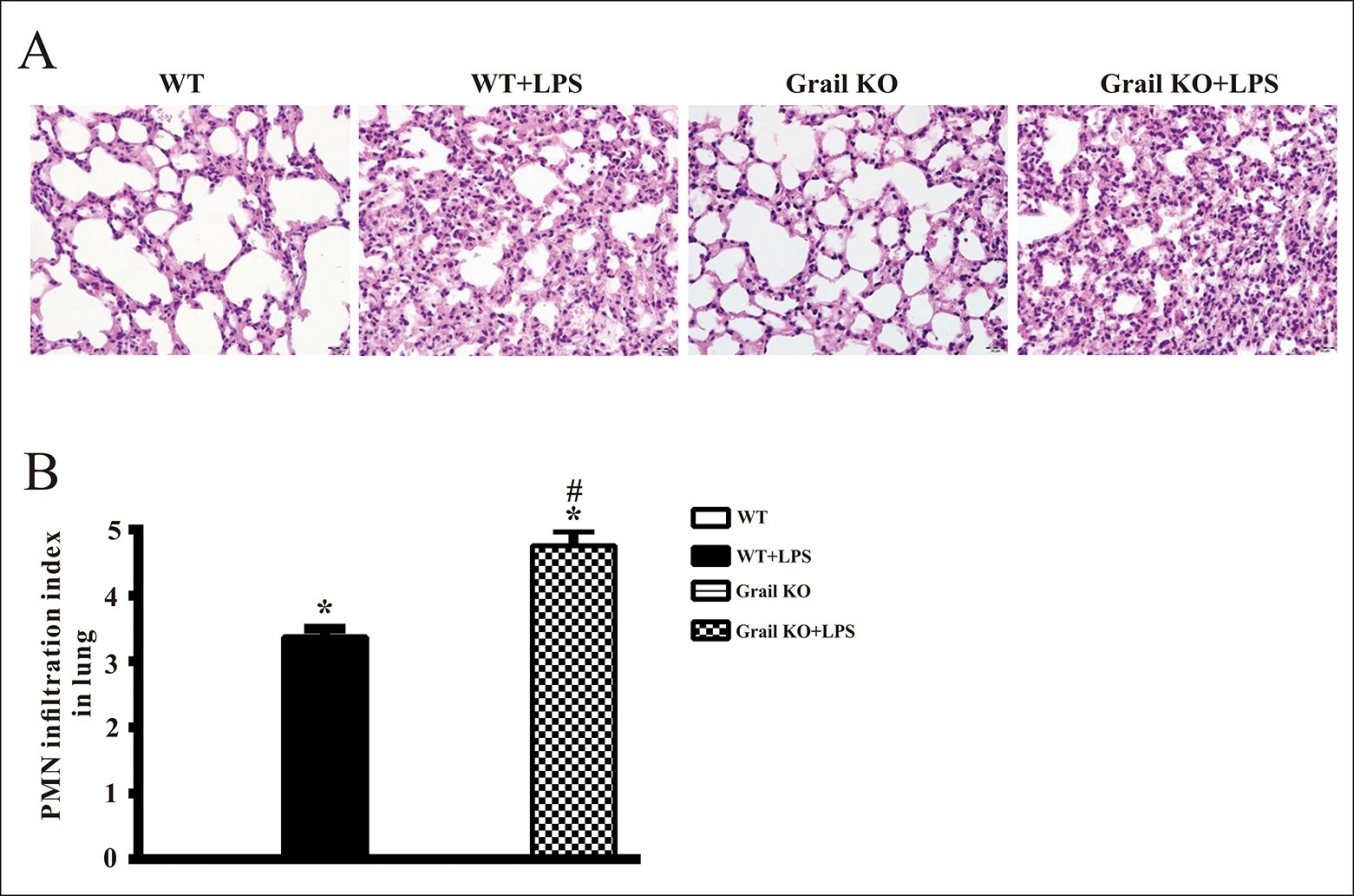
Effect of Grail deletion on lung histopathology in endotoxemic mice. Depicted are (A) representative histopathologic pictures and (B) polymorphonuclear neutrophil (PMN) infiltration index of lung tissue sections from mice treated with LPS. Data are shown for WT mice administered saline at time 0 (WT, n = 4), WT mice administered LPS at time 0 (WT+LPS, n = 4), Grail KO mice administered saline at time 0 (Grail KO, n = 4), and Grail KO mice administered LPS at time 0 (Grail KO+LPS, n = 4). The data are presented as mean ± SEM. A one-way ANOVA with a Newman-Keuls post hoc test was used to evaluate statistical significance. *P < 0.05, LPS versus without LPS; ^#^*P* < 0.05, Grail KO+LPS versus WT+LPS. Original magnification, ×400.

In the lungs of WT and Grail^−/−^ groups, light microscopy did not reveal neutrophil infiltration (Fig 5). However, LPS resulted in overt neutrophil infiltration into the lungs of WT and Grail^−/−^ mice (Fig 5). However, levels of PMN infiltration in the lungs of Grail-null endotoxemic mice were significantly higher than those in the lungs of WT endotoxemic mice (Fig 5).

### Effect of Grail deletion on MMP-9 expression in the lungs of endotoxemic mice

**Fig 6:**
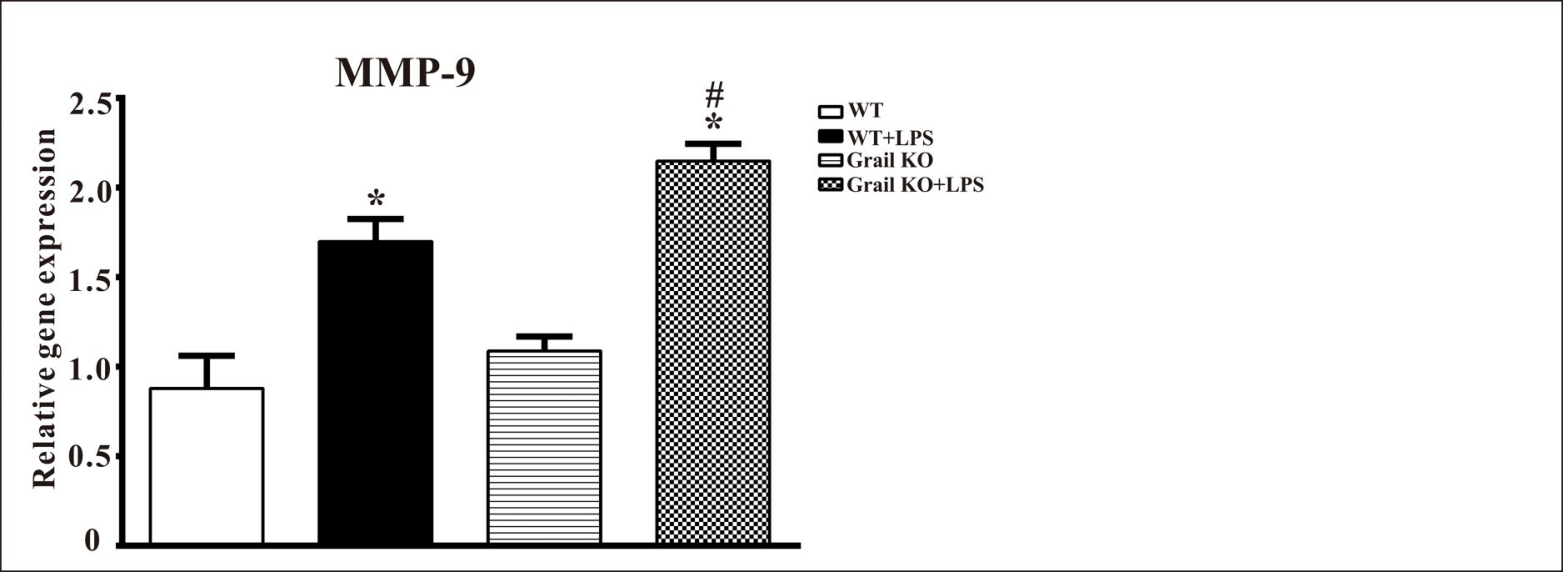
Effect of Grail deletion on *MMP-9* levels in the lungs of endotoxemic mice. Data are shown for WT mice administered saline at time 0 (WT, n = 3), WT mice administered LPS at time 0 (WT+LPS, n = 3), Grail KO mice administered saline at time 0 (Grail KO, n = 3), and Grail KO mice administered LPS at time 0 (Grail KO+LPS, n = 3). The data are presented as the mean ± SEM. A one-way ANOVA with a Newman-Keuls post hoc test was used to evaluate statistical significance. *P < 0.05, LPS versus without LPS; ^#^*P* < 0.05, Grail KO+LPS versus WT+LPS.

The levels of *MMP-9* mRNA were increased in the lung homogenates of WT and Grail^−/−^ mice treated with LPS (Fig 6). Grail deletion significantly augmented lung *MMP-9* levels in mice administered LPS (Fig 6). However, there was no significant difference in *MMP-9* levels between WT and Grail^−/−^ groups (Fig 6).

### Effect of Grail deletion on caspase 3 expression in the lungs of endotoxemic mice

**Fig 7:**
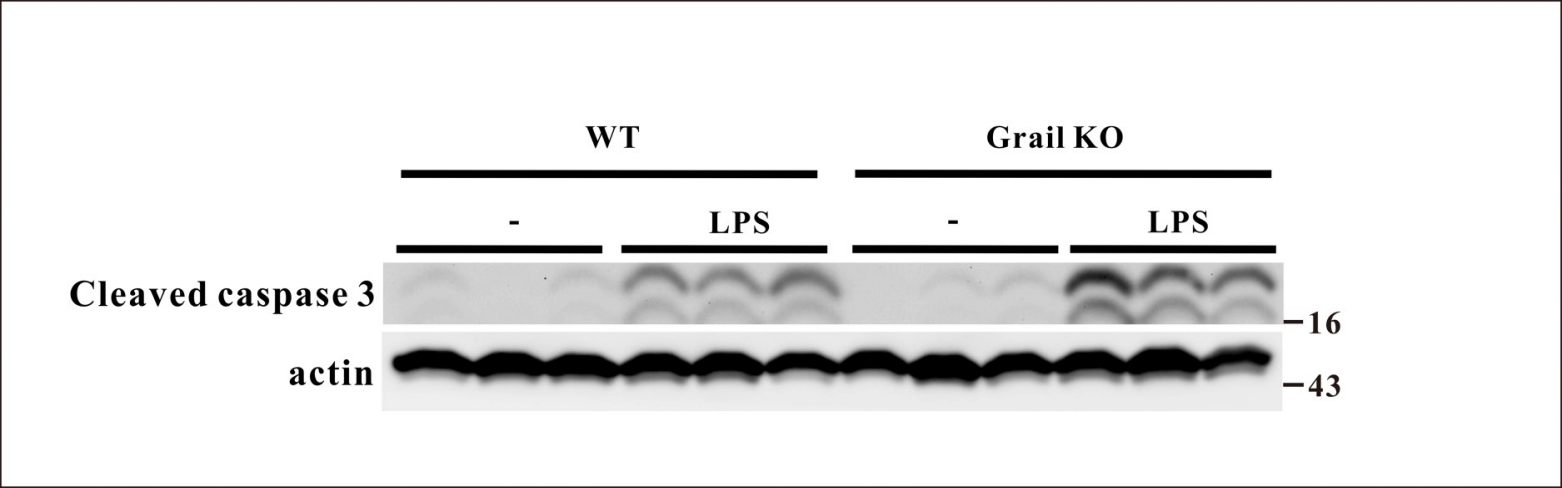
Effect of Grail deletion on caspase 3 expression in the lungs of endotoxemic mice. Data are shown for WT mice administered saline at time 0 (WT, n = 3), WT mice administered LPS at time 0 (WT+LPS, n = 3), Grail KO mice administered saline at time 0 (Grail KO, n = 3), and Grail KO mice administered LPS at time 0 (Grail KO+LPS, n = 3).

There was no significant difference in caspase 3 expression in the lungs between WT and Grail^−/−^ groups (Fig 7). The expression of caspase 3 in the lungs of WT and Grail^−/−^ mice was significantly upregulated after LPS administration (Fig 7). Moreover, Grail deletion significantly increased caspase 3 expression in the lungs of animals administered LPS (Fig 7).

### Effects of Grail deletion on body weight and hemodynamic parameters in endotoxemic mice

Body weight, SBP, and HR were not different among all groups at the beginning of the experiment (S2 Table). The injection of LPS caused significant decreases in body weight, SBP, HR, and blood glucose at 24 h in both WT and Grail^−/−^ mice (S2 Table). However, there were no significant differences in body weight and hemodynamic parameters between WT+LPS and Grail^−/−^+LPS groups (S2 Table).

## Discussion

Our *in vitro* and *ex vivo* studies showed that loss of Grail enhances the expression of pro-inflammatory cytokines in macrophages treated with LPS. Furthermore, LPS-mediated macrophage hyperactivation was attenuated by Grail overexpression. Based on *in vivo* studies, wild-type mice that received LPS exhibited circulatory failure, hypoglycemia, and organ dysfunction. Increased levels of *MMP-9* and caspase 3 as well as PMN infiltration were found in the lungs of endotoxemic mice. However, levels of *MMP-9*, caspase 3, and PMN infiltration in the lungs of Grail-null endotoxemic mice were significantly higher than those in the lungs of endotoxemic mice. Deletion of Grail also exacerbated organ damage in endotoxemic animals. Thus, these results suggest that Grail contributes to the regulation of M1-macrophages during sepsis and could protect against organ injury through the attenuation of over-inflammation and apoptosis in the lung of endotoxemic mice.

Macrophages are classified into two distinct phenotypes based on the immune microenvironment. M1 macrophages produce pro-inflammatory cytokines (e.g. TNF-α, IL-1β, and IL-6) and overexpress iNOS, whereas M2 macrophages secrete anti-inflammatory cytokines (e.g. IL-10) [7]. In the early phase of sepsis, LPS was previously shown to stimulate Toll-like receptors in macrophages, which undergo M1 differentiation to produce pro-inflammatory cytokines and overexpress iNOS [8]. Our data showed that LPS upregulated the expression of TNF-α, IL-1β, and IL-6 in J774A, RAW264.7, and primary peritoneal macrophages. Deletion of Grail augmented the expression of these pro-inflammatory cytokines (TNF-α, IL-1β, and IL-6) in macrophages treated with LPS. In addition, the levels of M1 macrophage marker iNOS in primary peritoneal macrophages of Grail KO mice were higher than WT mice in the presence of LPS. We further demonstrated that Grail overexpression results in the attenuation of pro-inflammatory signaling in LPS-stimulated macrophages. Thus, these data indicate that Grail is crucial for the regulation of inflammation triggered by activated macrophages during sepsis.

Multiple organ dysfunction and circulatory failure in sepsis are caused by systemic inflammation after exposure to microbial toxins [15, 16]. LPS recognition by Toll-like receptors in macrophages drives several cellular pathways involved in inflammation [4, 5]. The uncontrolled release of pro-inflammatory cytokines from activated macrophages has been proposed to be a major factor related to the outcome of sepsis [17, 18]. In this study, we found that IL-1β, TNF-α, IL-6, COX-2, and iNOS expression in the primary peritoneal macrophages of Grail-deficient mice was higher than that in normal mice in the presence of LPS. Grail deletion also resulted in increased levels of MMP-9 and PMN infiltration in the lungs of endotoxemic animals. Moreover, organ injury and apoptosis caused by the injection of LPS were significantly exacerbated by Grail deletion. Therefore, these results suggest the severity of inflammation corresponds to Grail expression based on our *ex vivo* and *in vivo* studies, which implies that Grail protects against organ injury and apoptosis by attenuating hyperinflammation during sepsis.

Recently, Aziz et al. have revealed that Grail expression is induced in CD4 T cells and leads to the inhibition of T cell proliferation during the progression of sepsis [14]. However, our data showed that Grail protein expression was obviously reduced in macrophage cells after LPS treatment. We suggest the downregulation of Grail in LPS-treated macrophages was regulated by post-translational modifications such as ubiquitination. Thus, Grail could exert many cellular functions by regulating various mechanisms in different cell types under conditions of stress.

Several transcription factors participate in the regulation of sepsis progression by modulating immunity and the inflammatory response [19–21]. Peroxisome proliferator-activated receptor γ (PPARγ) is considered a promising target for sepsis treatment [22, 23]. Our recent study showed that Grail can regulate PPARγ-dependent fat cell differentiation [13]. Thus, the correlation between Grail and PPARγ during the modulation of sepsis development and the possible associated molecular mechanisms is worth further study. In addition, our previous study demonstrated that Grail can mediate p53-dependent cell cycle arrest and apoptosis by targeting it for degradation. The tumor suppressor p53 triggers cell cycle arrest, apoptosis and senescence in response to different types of stress [24, 25]. Recent studies show that p53 status is involved in sepsis-induced cell death and the systemic inflammatory response *in vitro* and *in vivo* [26–28]. However, the activity and function of p53 in Grail-expressing macrophages are still unclear. We thus need further to characterize the interaction between Grail and p53 during sepsis-associated macrophage activation.

Taken together, these findings indicate that Grail contributes to the attenuation of hyperinflammation triggered by activated macrophages and prevents organ injury and apoptosis in endotoxemic mice. Although the molecular mechanisms associated with the function of Grail during sepsis require further investigation, the present study suggests that Grail signaling could be the potential therapeutic target for infectious diseases.

## Supporting information

S1 Table The primer sequences of Grail, IL-1β, TNF-α, IL-6, COX-2, and MMP-9. (DOCX)

S2 Table Effects of Grail deletion on body weight and hemodynamic parameters in endotoxemic mice. Depicted are changes in body weight, systolic blood pressure (SBP), heart rate (HR), and blood glucose of animals. WT, WT mice administered saline at time 0 (n = 8); WT+LPS, WT mice administered LPS at time 0 (n = 10); Grail KO, Grail KO mice administered saline at time 0 (n = 8); Grail KO+LPS, Grail KO mice administered LPS at time 0 (n = 11). Data are shown as mean ± SEM. *P < 0.05, LPS versus without LPS; ^†^*P* < 0.05, Grail KO+LPS versus WT+LPS.
(DOCX)
