# Supplementary material for: Macrophage expression of E3 ubiquitin ligase Grail protects mice from lipopolysaccharide-induced hyperinflammation and organ injury
Source: PLoS One. 2018 Dec 20;13(12):e0208279. doi: 10.1371/journal.pone.0208279 (PMC6301572; doi:10.1371/journal.pone.0208279)
Supplement: S1 Table — (DOCX) [file pone.0208279.s001.docx]

**S1 Table. The primer sequences of Grail, IL-1β, TNF-α, IL-6, COX-2, and MMP-9.**

| **Primers** | **Forward sequence** | **Reverse sequence** |
| --- | --- | --- |
| **Grail** | *5’-GCGTCTGGAGCCGTCATCTTTA-3’* | *5’-GGGCCATGTTTTTTCCCTACTTCTAT-3’* |
| **IL-1β** | *5’-CCCAAAAGATGAAGGGCTGC-3’* | *5’-AAGGTCCACGGGAAAGACAC-3’* |
| **COX-2** | *5’-CTGCGCCTTTTCAAGGATGG-3’* | *5’-ACATCATCAGACCAGGCACC-3’* |
| **TNF-α** | *5’-TAGCCCACGTCGTAGCAAAC-3’* | *5’-GATAGCAAATCGGCTGACGG-3’* |
| **MMP9** | *5’-ATAGAGGAAGCCCATTACAGG-3’* | *5’-GTGTACACCCACATTTGACG-3’* |
| **B2M** | *5’-ACCGTCTACTGGGATCGAGA-3’* | *5’-TGCTATTTCTTTCTGCGTGCAT-3’* |
| **GAPDH** | *5’-TTCACCACCATGGAGAAGGC-3’* | *5’-GATGGCATGGACTGTGGTC-3’* |
