# Supplementary material for: Macrophage expression of E3 ubiquitin ligase Grail protects mice from lipopolysaccharide-induced hyperinflammation and organ injury
Source: PLoS One. 2018 Dec 20;13(12):e0208279. doi: 10.1371/journal.pone.0208279 (PMC6301572; doi:10.1371/journal.pone.0208279)
Supplement: S2 Table — Depicted are changes in body weight, systolic blood pressure (SBP), heart rate (HR), and blood glucose of animals. WT, WT mice administered saline at time 0 (n = 8); WT+LPS, WT mice administered LPS at time 0 (n = 10); Grail KO, Grail KO mice administered saline at time 0 (n = 8); Grail KO+LPS, Grail KO mice administered LPS at time 0 (n = 11). Data are shown as mean ± SEM. *P < 0.05, LPS versus without LPS; †P < 0.05, Grail KO+LPS versus WT+LPS. (DOCX) [file pone.0208279.s002.docx]

|  | **0 h** | **24 h** |
| --- | --- | --- |
| Body weight (g) |  |  |
| WT | 27.5 ± 0.9 | 27.8 ± 1.0 |
| WT+LPS | 27.6 ± 0.6 | 25.4 ± 0.7* |
| Grail KO | 28.1 ± 0.5 | 28.5 ± 0.7 |
| Grail KO +LPS | 28.0 ± 0.7 | 25.2 ± 0.5* |
| SBP (mmHg) |  | |
| WT | 115 ± 4 | 120 ± 3 |
| WT+LPS | 129 ± 4 | 80 ± 11* |
| Grail KO | 126 ± 3 | 131 ± 3 |
| Grail KO+LPS | 123 ± 5 | 69 ± 4* |
| HR (beats/min) |  | |
| WT | 737 ± 13 | 739 ± 14 |
| WT+LPS | 692 ± 22 | 511 ± 27* |
| Grail KO | 701 ± 15 | 761 ± 10 |
| Grail KO+LPS | 670 ± 30 | 476 ± 17* |
| Blood glucose (mg/dL) |  | |
| WT | - | 256 ± 21 |
| WT+LPS | - | 78 ± 16* |
| Grail KO | - | 232 ± 12 |
| Grail KO+LPS | - | 55 ± 7* |

**S2 Table. Effects of Grail deletion on body weight and hemodynamic parameters in endotoxemic mice**. Depicted are changes in body weight, systolic blood pressure (SBP), heart rate (HR), and blood glucose of animals. WT, WT mice administered saline at time 0 (n = 8); WT+LPS, WT mice administered LPS at time 0 (n = 10); Grail KO, Grail KO mice administered saline at time 0 (n = 8); Grail KO+LPS, Grail KO mice administered LPS at time 0 (n = 11). Data are shown as mean ± SEM. **P* < 0.05, LPS versus without LPS; ^†^*P* < 0.05, Grail KO+LPS versus WT+LPS.
